# Coupled evolutionary rates shape a Hawaiian insect-symbiont system

**DOI:** 10.1186/s12864-025-11514-z

**Published:** 2025-04-03

**Authors:** Patrick H. Degnan, Diana M. Percy, Allison K. Hansen

**Affiliations:** 1 Department of Microbiology and Plant Pathology, University of California, Riverside, CA USA; 2 Department of Botany, University of British Columbia, Vancouver, BC Canada; 3 Department of Entomology, University of California, Riverside, California USA

**Keywords:** Galling, Species radiation, Triozidae, Psylloidea, Hemiptera, Mitogenome, *Carsonella*

## Abstract

**Background:**

The Hawaiian *Pariaconus* psyllid radiation represents a unique system to study the co-evolution of nuclear, mitochondrial, and endosymbiont genomes. These psyllids, which diversified across the Hawaiian Islands during the last 3–3.5 million years vary with their ecological niches on their plant host ‘Ōhi’a lehua (*Metrosideros polymorpha*) (free-living, open-gall, and closed-gall lifestyles) and harbor one to three beneficial bacterial endosymbionts. Co-evolutionary studies of other multi-endosymbiont insect systems have shown decoupled rates of sequence evolution between mitochondria and endosymbionts. Here we examine the evolutionary trends in *Pariaconus* psyllids, their mitochondria and their endosymbionts to determine if they fit this paradigm.

**Results:**

We sequenced a new *Carsonella* genome from the *ohialoha* species group (closed-gall, one symbiont), revealing a remarkable degree of gene conservation between two of the most divergent species from this diverse species group that has dispersed across multiple islands. Further, despite the rapid radiation of psyllid species, we observed complete synteny among mitochondrial genomes from all six *Pariaconus* species in this study, suggesting the preservation of genome structure due to strong purifying selection. Phylogenetic analyses of the nuclear, mitochondrial, and endosymbiont genomes across these six *Pariaconus* species revealed correlated rates of substitutions, contrary to prior reports of decoupling between mitochondrial and endosymbiont genomes in other insect systems with multiple symbiont partners. Finally, we found that free-living psyllids with three symbionts exhibited elevated mutation rates (~ 1.2–1.6x) across all genomes and elevated rates of fixation of nonsynonymous substitutions in the insect nuclear genome and one of the endosymbionts.

**Conclusions:**

This study highlights the interplay between ecological diversification and genomic evolution in *Pariaconus*. Further, these data indicate that multiple endosymbiont partners alone are not sufficient to result in decoupling rates of sequence evolution. Future work on basal members of this species radiation will refine our understanding of the mechanisms shaping this dynamic insect-symbiont system and its implications for genome evolution.

**Supplementary Information:**

The online version contains supplementary material available at 10.1186/s12864-025-11514-z.

## Background

In animals, multiple genomes—nuclear, mitochondrial, and symbionts—can coexist within a single organism, each evolving through distinct mechanisms and processes. The nuclear genome, generally inherited biparentally, is the primary source of genetic material and undergoes mutation, recombination, selection, and genetic drift over generations [1]. In contrast, the mitochondrial genome is maternally inherited and evolves more rapidly due to a higher mutation rate, with selective pressures typically acting to maintain its efficiency of cellular respiration [2]. Meanwhile, symbionts, such as endosymbiotic bacteria, can experience vertical and/or horizontal transmission and their genomes are shaped by co-evolutionary dynamics with their host with beneficial and/or parasitic consequences [3]. While these genomic systems evolve independently, they can be interconnected, with evolutionary processes such as mitonuclear co-evolution and host-symbiont interactions influencing their genetic trajectories [4]. The interplay between these structurally and functionally diverse genomes can lead to a dynamic evolutionary landscape, where selective pressures may align or conflict, shaping their genetic and functional integration with the host organism.

These evolutionary dynamics can become unpredictable when multiple endosymbiotic bacteria reside in a single animal host. Previous studies report that despite similar vertical transfer, mutational biases, and lack of recombination among insect beneficial symbionts and mitochondria their rates of evolution are not always correlated [5, 6]. One factor suspected of contributing to this was the presence of more than one symbiont [6], which may result in natural selection affecting each symbiont differently. Examining this phenomenon in additional systems has the potential to determine how common the decoupling of natural selection among co-inherited intracellular genomes is as a function of the number of genomes present. Interestingly, a recent study revealed that Hawaiian *Pariaconus* psyllid species have one, two, or three distinct, co-evolved bacterial endosymbiont lineages that contribute to their nutrition and this is associated with their ecological niches of free-living, open-gall, and closed-gall, respectively [7]. This genus has diversified across the Hawaiian archipelago for several million years, resulting in over 36 species that all depend on a single endemic host-plant, ‘Ōhi’a lehua (*Metrosideros polymorpha*) [8]. This level of diversification for a monophyletic lineage on a single host-plant species is rare not only for psyllids but for other insect herbivores [9–11]. Likely contributors to *Pariaconus* speciation have been the high degree of polymorphism in *M. polymorpha* [12] and the exploitation of different ecological niches on their host-plant (e.g., galling type) [8].

**Fig. 1:**
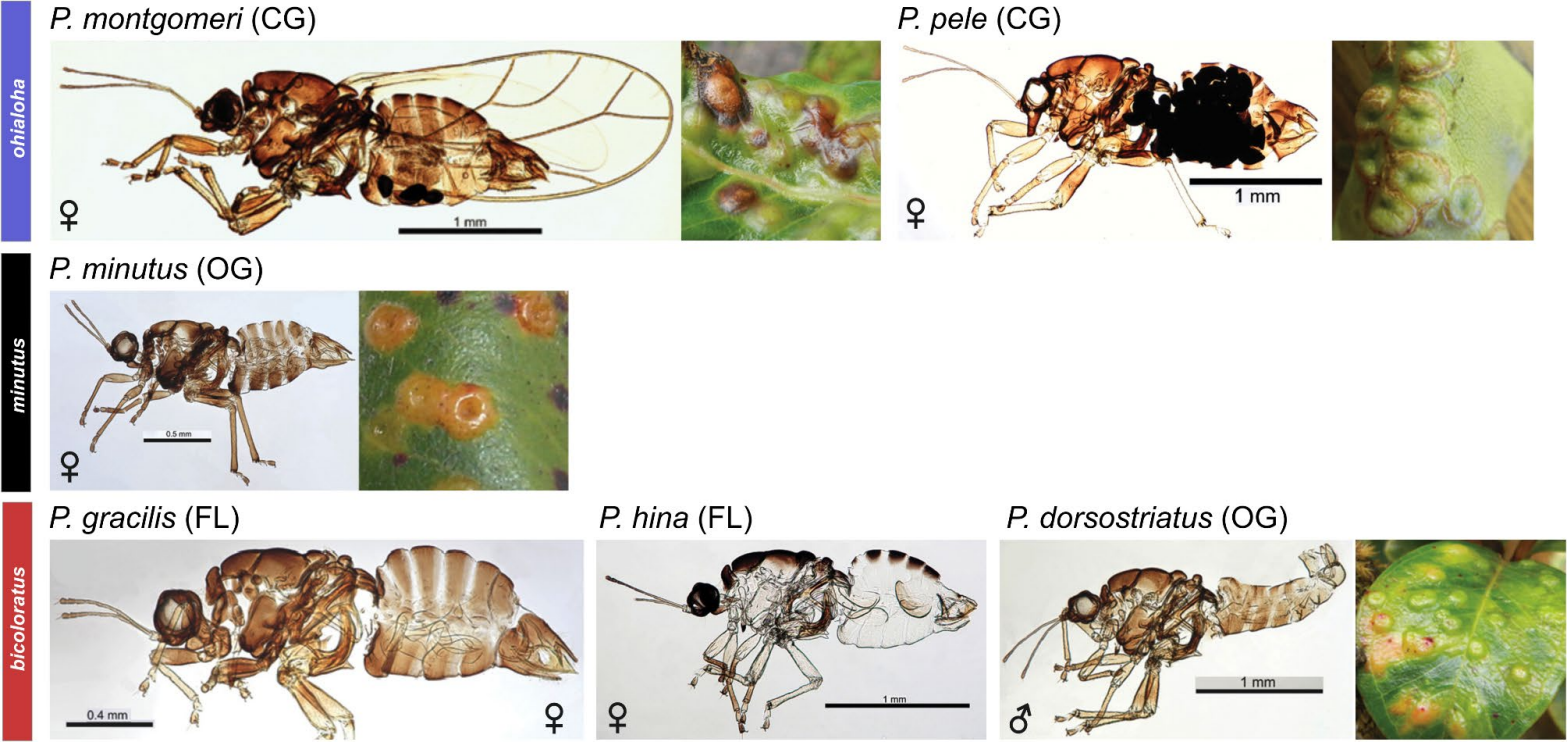
Hawaiian *Pariaconus* psyllids. Lateral views of representative Hawaiian *Pariaconus* psyllid species sampled herein. Species are organized by species group *ohialoha*, *minutus* and *bicoloratus*. Each insect species is labeled and includes both a scale bar and sex of the imaged animal. Open-gall (OG) and closed-gall (CG) insects are paired with images of their corresponding gall formations. All images from Percy 2017 (Creative Commons License)

As such, this insect-symbiont system provides an ideal opportunity to investigate the evolutionary forces shaping these diverse genomes. We investigate whether the evolutionary trends in *Pariaconus* psyllids and their endosymbionts follow the coupled sequence evolution pattern observed for single-endosymbiont insect systems [5, 13, 14] or the decoupled pattern for some multi-endosymbiont systems [5, 6]. To do so, we analyzed metagenomic data generated here and in Hansen et al. [7] for additional insights into the comparative rate of evolution of *Pariaconus*’s nuclear, mitochondrial, and endosymbiont genomes. Here, we have sequenced and assembled an additional endosymbiont genome (*Carsonella*) as well as partial nuclear and complete mitochondrial genomes from six divergent *Pariaconus* psyllid species. These psyllids represent three out of the four species groups (*bicoloratus*, *minutus*, and *ohialoha*) within *Pariaconus.* Our sampling design enabled us to evaluate the rate of evolution among species pairs that vary in the number of symbionts present; one (*P. pele vs. P. montgomeri*), two (*P. minutus vs. P. dorsostriatus*) or three (*P. gracilis vs. P. hina*) [7]. The differences in number of symbiont partners also coincide with differences in host lifestyle, forming closed-galls, open-galls or free-living, respectively (Fig. 1). Employing likelihood estimators of synonymous and nonsynonymous substitution and divergence estimates inferred from the geological record of the Hawaiian Islands, we compare substitution rates within and between these associated genomes. Ultimately, we report strong evidence of correlated rates among the host, mitochondrial and symbiont genomes in this recently derived species radiation regardless of symbiont number or lifestyle. Further, we find that the free-living psyllids with three symbionts have evidence of elevated mutation rates influencing their nuclear, mitochondrial, and symbiont genomes compared to the other two psyllid galling groups examined.

## Methods

### Sampling and identification

Field collections by Diana Percy (DP) in 2014 were made using a sweep-net or aspirating directly from the leaves of the host-plant *M. polymorpha* in the field (see Percy 2017 for additional details regarding sampling and species identification). Specimens were transferred directly to vials in 95% ETOH for DNA sequencing or RNAlater (Qiagen) for RNA sequencing and stored at -20 °C. The eight psyllid specimens were identified by DP as *Pariaconus gracilis* (Crawford, 1918) f. *gracilis*, *Pariaconus hina* Percy, 2017 f. *ovostriatus*, *Pariaconus dorsostriatus* Percy, 2017 f. *communis*, *Pariaconus minutus* (Crawford, 1918) f. *minutus*, *Pariaconus pele* Percy, 2017 f. *pele* [1], *Pariaconus pele* Percy, 2017 f. *pele* [2], *Pariaconus montgomeri* Percy, 2017 f. *montgomeri* and *Pariaconus montgomeri* Percy, 2017 f. *paliuliensis*. Location and metadata for each sample are provided in Table 1. RNA extraction used entire specimens following Misof et al. [15], while non-destructive DNA extractions used whole individual psyllid specimens with the Qiagen Blood and Tissue Kit (Qiagen) or QIAamp UCP DNA Micro Kit (Qiagen). All DNA was consumed during library preparation, however, voucher specimens from non-destructive extractions were preserved in ethanol (70–85%) and retained in DPs DMPC collection at the University of British Columbia. Contact person for collection is DP (diana.percy@ubc.ca). DMPC Voucher numbers for the eight specimens are as follows: DMP-Hi61-14-gracilis-25-23Oct14, DMP-Hi16-14-dorsostriatus-4-09Sep14, DMP-Hi53-14-hina-16-22Oct14, DMP-Hi10-14-minutus-9-04Sep14, DMP-Hi17-14-pele-7-09Sep14, DMP-Hi24-14-pele-1-11Sep14, DMP-Hi49-14MCf1_7A-montgomeri-2014 and DMP-Hi52-14-montgomeri-2015.

**Table 1:** Hawaiian psyllid sample collection data

Coll. Code	Species (tube)	Lat. *N*	Long. W	Alt. (m)	Date collected
DMP-Hi61-14-gracilis	*P. gracilis* (25)	21.5028	-158.1483	1201	4-Jul-14
DMP-Hi16-14-dorsostriatus	*P. dorsostriatus* (4 A)	19.5516	-155.2308	1148	6-Mar-14
DMP-Hi53-14-hina	*P. hina* (16 A)	20.8106	-156.2398	1308	3-Jul-14
DMP-Hi10-14-minutus	*P. minutus* (9B)	20.0853	-155.6778	1270	5-Mar-14
DMP-Hi17-14-pele	*P. pele* (7 A)	19.5522	-155.2310	1133	6-Mar-14
DMP-Hi24-14-pele	*P. pele* (1)	19.2301	-155.7818	1755	7-Mar-14
DMP-Hi49-14-montgomeri	*P. montgomeri* (7 A)	20.9339	-156.6132	921	2-Jul-14
DMP-Hi52-14-montgomeri	*P. montgomeri*	20.8099	-156.2501	1306	3-Jul-14

### Transcriptome and metagenome sequencing

For one of the *P. montgomeri* samples (DMP-Hi52-14-montgomeri-2015) messenger RNA libraries were generated from one microgram of high-quality total RNA from 10 psyllid adult whole-bodies collected by DP. The RNA sample was then processed with the TruSeq Stranded RNA Sample Preparation Kit (Illumina, San Diego, CA). Paired-end 100 bp reads were generated on an Illumina HiSeq2500 with TruSeq SBS sequencing kits version 4 at the University of Illinois at Urbana-Champaign sequencing facility. For the remaining samples collected by DP, DNA Illumina libraries were generated with the New England Biolabs (Ipswich, MA) FS DNA Library Prep Kit (E7805, E6177). Sequencing of the libraries were carried out on the NextSeq2000 with paired-end 300 bp reads by the University of California, Riverside Genomics Core Facility. Briefly, all read data were then quality trimmed by Trimmomatic v.0.36 (ILLUMINACLIP: TruSeq3_PE.fa:2:30:10 LEADING:3 TRAILING:3 SLIDINGWINDOW:4:15 MINLEN:50) [16], initial assemblies were completed with default parameters in MEGAHIT (v1.2.9) [17] and reads were mapped to the contigs with Bowtie2 (v2.5.3) with default parameters to generate read coverage information [18]. See detailed description of bioinformatic steps at https://github.com/phdegnan/Evolution-in-Pariaconus for additional details.

### Carsonella genome assembly and annotation

A single putative *Carsonella* contig (*Carsonella-Pm*) in the *P. montgomeri* Hi49-14MCf1_7A (Hi49) metagenome was detected with BLASTn using an existing *Carsonella* genome from *Pariaconus pele* (GCA_044442925.1) as a query. Contig edges were first compared using BLASTn for possible sequence overlap and then compared to related *Carsonella* genomes. This produced a proposed circular junction for the *Carsonella* replicon which was then confirmed based on the detection of continuous, overlapping paired end reads among the Illumina sequencing data spanning this sequence. Automatic annotations for the *Carsonella-Pm* genome were predicted with PROKKA (v1.14.5; --kingdom Bacteria --rfam) [19]. Functional annotations were confirmed with HMMSCAN (hmmer.org) searches using trusted cutoffs (--cut_tc) against the PFAMs [20] and TIGRFAMs [21] databases. Potentially missing or mutationally inactivated genes were manually confirmed using BLASTx. *Carsonella-Pm* and related genomes were aligned using the mauveAligner algorithm with its default parameters in the Mauve application (snapshot_2015-02-25) [22].

### Mitochondrial genome assembly and annotation

Putative mitochondrial contigs were detected with BLASTn using the existing *Pariaconus pele* mitogenome (NC_038138) as a query. Reads for the relevant contigs were extracted and reassembled with Spades (v3.15.5; --isolate) [23]. Contig edges were then manually inspected, and circularization of the replicons was confirmed with the Illumina sequencing data if possible. Automatic annotations for each individual genome were predicted with PROKKA (v1.14.5; --kingdom Mitochondria --rfam) [19] then manually compared to NC_038138 and visualized in SnapGene (v5.2.1). Annotations for short ORFs and spurious, predicted tRNAs in non-canonical locations were removed. Start and stop positions of remaining tRNAs and ORFs were adjusted to match NC_038138. Note, short read alignments were inspected and no base-call errors in these regions were detected. All annotations were evaluated and confirmed by NCBI staff during the submission process and prior to their release. Final estimates of read coverage were determined with Bowtie2 (v2.5.3) [18] and the mitogenomes were aligned using Mauve [22].

### Identification of *Pariaconus* nuclear core genes

Single copy BUSCOs previously identified from whole genome chromosome sequencing of *Bactericera cockerelli* (*n =* 1,677) [24], which belongs to the same psyllid family (Triozidae) as *Pariaconus*, were used as queries in tBLASTn searches to identify potential metagenome assembled contigs encoding orthologous genes (-evalue 1e-10 -max_target_seqs 15 -outfmt “6 std qlen slen”). The results were filtered for the best matches with ≥ 50% percent of the query gene aligning to a single metagenome contig and with ≥ 60% amino acid identity. Exonerate (v2.4.0) was then run on the DNA metagenomes for the subset of single copy BUSCOs detected in all 7 samples (--refine region --model protein2genome --showtargetgff --percent 25). Predicted exons were extracted for each gene using GFFREAD (CUFFLINKS v2.2.1), then merged into a predicted transcript using a custom script. We previously analyzed the *P. montgomeri* transcriptome assembly (DMP-Hi52-14-montgomeri-2015) with BUSCO and its single copy BUSCOs were directly compared to those of *B. cockerelli* using BLASTp [7]. Subsequent alignments identified a high degree of fidelity between *P. montgomeri* assembled transcripts and those identified by Exonerate for the other species.

### Phylogenetic reconstruction

Whole genome phylogenetic reconstructions were generated using predicted protein coding sequences for each dataset (host nuclear, mitochondrial, *Carsonella*). This was performed with OrthoVenn3 [25]. First, amino acid translations were generated for the host nuclear gene transcripts, and the mitochondrial and symbiont genes with the EMBOSS (v6.6.0) tool TRANSEQ. Additional published mitochondrial genomes from Triozidae [ 26, 27] were retrieved from NCBI and analyzed along with the deposited *P. pele* sequence NC_038138 and the seven new mitogenomes sequenced here. Furthermore, the *Carsonella-Pm* genome (sequenced here) was analyzed with the six previously sequenced *Carsonella* genomes from *Pariaconus* [7]. OrthoVenn3 uses the approximately-maximum-likelihood tree estimation tool FastTree2 [28] to construct a phylogenetic tree based on the concatenated alignment of all single copy shared protein sequences using the JTT + CAT model of evolution. Gene Ontology annotations for the host nuclear genes were also retrieved from the OrthoVenn3 analysis to annotate all the shared orthologs with GO terms. In addition, the concatenated protein alignments from OrthoVenn3 for the *Pariaconus* psyllid, mitochondrial and *Carsonella* datasets were used to independently estimate phylogenies using ModelTest-ng (v0.2.0) [29] and RAxML-ng (v1.2.0) [30]. Optimal models were chosen using AIC (JTT + I + G4 + F, MTMAM + I + G4 + F, and JTT-DCMUT + I + G4 + F respectively) and RAxML-ng was run using the default parameters including 200 bootstrap replicates.

In addition, published *Pariaconus* CytB and COI sequences [8, 31] were retrieved from NCBI (Supplemental Table 4) and aligned with the same gene regions from NC_038138 and the seven new mitogenomes using Muscle (v3.8.1551) [32]. The CytB and COI alignments were then analyzed by approximately-maximum-likelihood tree estimation with FastTree2 (v2.1.11) [28].

### Estimating rates of gene evolution

Transcripts for each orthologous host, mitochondrial, *Carsonella*,* Makana* (*Morganella*-like), and *Malihini* (*Dickeya*-like) gene were aligned based on their amino acid translations with MUSCLE (v5.1.linux64) [32]. Subsequently, gaps and stop codons were removed from each transcript alignment before rates of synonymous substitutions per synonymous site (dS) and nonsynonymous substitutions per nonsynonymous site (dN) were estimated with CodeML (v4.9) using the strategies to detect positive selection with heterogenous dN/dS rates across sites (see [33]). Clustalw (v2.1) was used to estimate pairwise distances among the mitochondrial genes [34].

To estimate absolute substitution rates we determined the ages for the internal nodes of the OrthoVenn3 generated *Carsonella* whole genome phylogeny using r8s (v1.8) [35]. Ages were estimated by ‘fixing’ the age of the divergence between the *bicoloratus* and *minutus* clades at 3.5 or 3.0 MYA based on the rate of divergence identified in Percy [8], phylogenetic topology [27], and island ages. Node dates were then used as divisors to determine the average rates of dS and dN per year (dS/t and dN/t). Genes with saturated dS values (dS ≥ 3) were excluded unless otherwise indicated. Statistical analysis of dS/t, dN/t and dN/dS were performed in JMP v17. Given the range in gene sample sizes and presence of non-normally distributed data, nonparametric tests were used when possible, including pairwise comparisons using the Wilcoxon Each Pair Method and Spearman’s rho.

## Results

### *Carsonella* of *P. montgomeri*

**Fig. 2:**
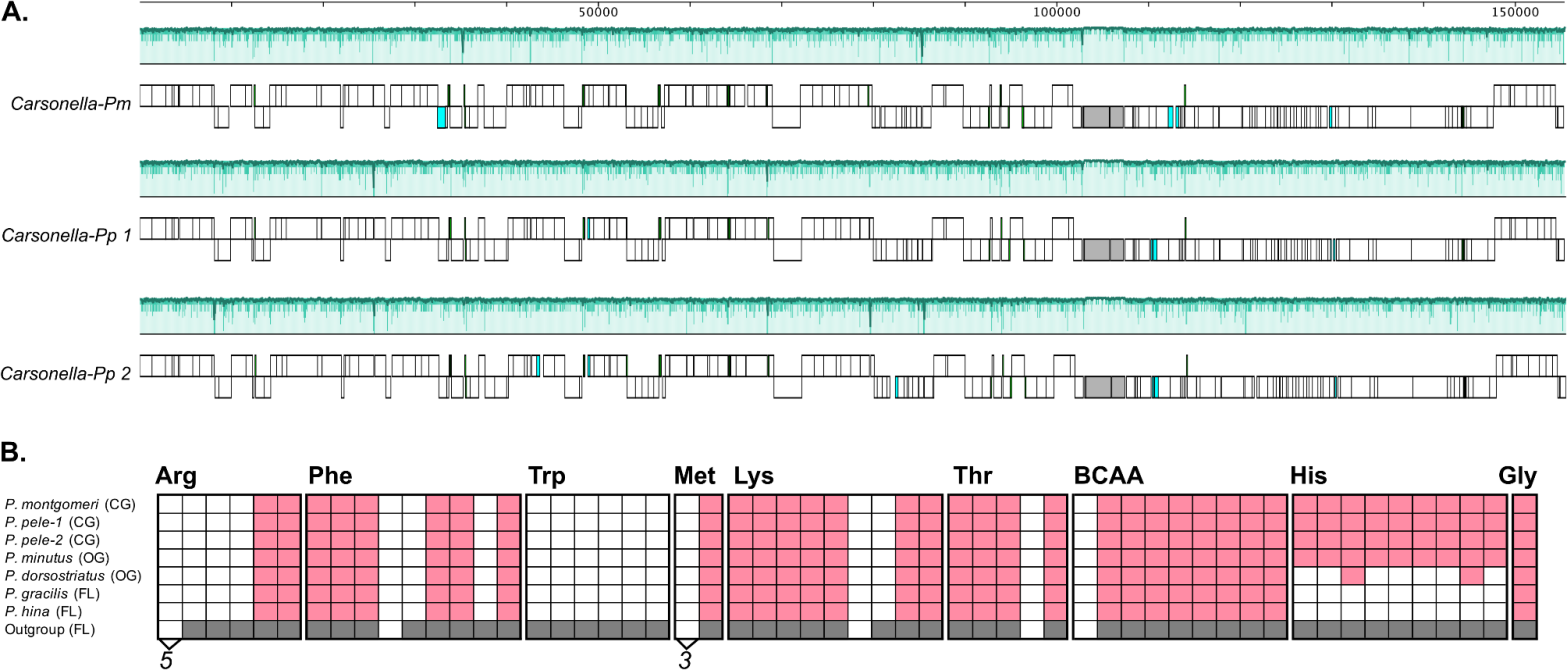
Comparative analysis of *Pariaconus Carsonella* genomes. (**A**) Whole genome alignment of *Carsonella-Pm* with previously sequenced *Carsonella-Pp* genomes exhibits complete genome synteny. Scaled schematic of genes is shown according to the scale bar in base pairs. Ribosomal genes are shown in grey and putative pseudogenes are shown in light blue. Multi-color shaded plot above each genome indicates the average NT similarity to the other genomes (range = 50–100%) as generated by Mauve. (**B**) Histidine biosynthesis genes are only detected in *Carsonella* genomes from galling *Pariaconus* psyllids. Gene presence matrix for genes involved in amino acid biosynthesis pathways (red). White cells indicate the absence of a required gene, or in the case of arginine and methionine biosynthesis the absence of multiple genes, which is indicated by a number below cells. Genes present in the free-living Triozidae outgroup (*B. cockerelli*) are shown in grey

The metagenome of *P. montgomeri* was sequenced resulting in 87 million raw read pairs and ultimately resulting in the assembly of the complete 155 kbp *Carsonella-Pm* genome (Supplemental Table 1). *Pariaconus montgomeri* is an early branching species of the closed-gall, one symbiont, *ohialoha* group of *Pariaconus* psyllids [27]. Comparisons with the previously sequenced *Carsonella* genomes from *P. pele*, a more recently derived member of the *ohialoha* group [7], reveal the expected collinearity commonly detected among obligate endosymbiont lineages (Fig. 2A). Further, there is pseudogenization of only three protein coding genes in *Carsonella-Pm* that appear intact in one or both of the *Carsonella-Pp* genomes. Conversely, there are three intact protein coding genes in *Carsonella-Pm* that are truncated to < 2/3 their expected lengths in both *Carsonella-Pp* genomes. This includes RplC 50 S ribosomal protein L3 and PrfB Peptide chain release factor RF2. Moreover, *Carsonella-Pm* has the same patterns of loss and retention of genes that are involved in the biosynthesis of 10 amino acids as observed in all previously sequenced *Pariaconus Carsonella* genomes (Hansen et al. 2024). This includes arginine, phenylalanine, tryptophan, methionine, lysine, threonine, isoleucine, leucine, and valine as well as the non-essential amino acid glycine (Fig. 2B). In addition, *Carsonella-Pm* encodes the genes for histidine biosynthesis (Fig. 2B). Genes for histidine biosynthesis, an ancestral *Carsonella* pathway found in outgroup psyllid family members of Triozidae, *B. cockerelli* [36] and *B. trigonica* [37], have so far only been detected among *Carsonella* symbionts of *Pariaconus* psyllids that form galls such as both *Carsonella-Pp* strains [7] and *Carsonella-Pm* sequenced here (Fig. 2B). Among the open gall forming *Pariaconus* psyllids sampled so far it is either fully intact (*Carsonella* of *P. minutus*) and or partially intact (*Carsonella* of *P. dorsostriatus*) with 2 of the 8 required genes (Fig. 2B).

It is possible that the broad gene content conservation detected between the *Carsonella-Pm* and *Carsonella-Pp* genomes underestimates their sequence divergence as their closed-gall psyllid hosts have diversified and speciated among the distinct Hawaiian Islands (Maui and Hawaii, respectively). Therefore, to further understand the evolution of *Pariaconus* psyllids, their galling habits, and their symbionts on the Hawaiian Islands we further analyzed metagenomic data here and in Hansen et al. [7] for additional insights into the comparative evolution of *Pariaconus*’s nuclear, mitochondrial, and endosymbiont genomes.

### Identification of Pariaconus mitochondrial genomes

**Fig. 3:**
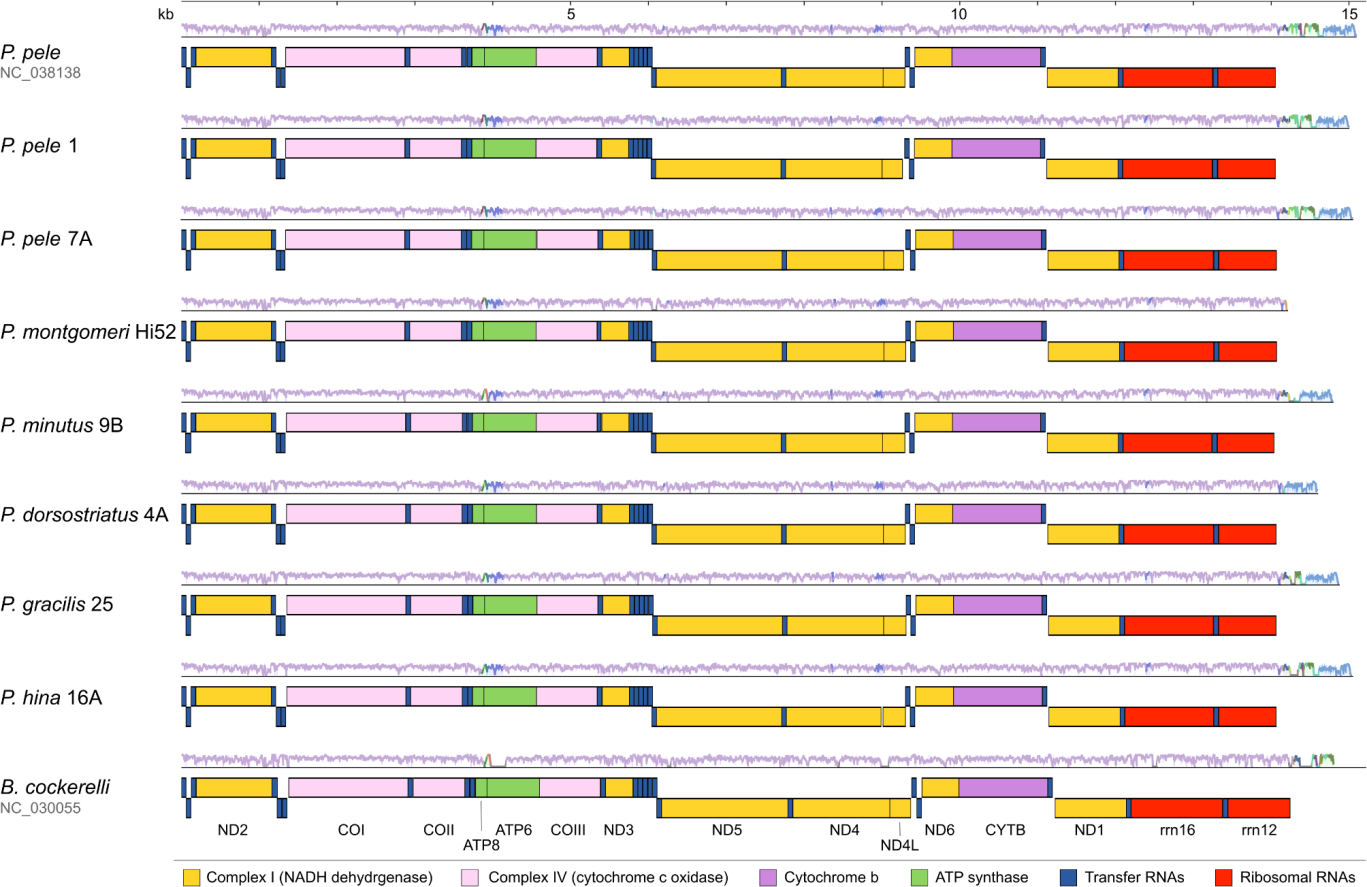
Whole mitogenome alignment of Hawaiian *Pariaconus* psyllids. Whole genome alignment shows complete synteny among Hawaiian *Pariaconus* psyllid mitochondrial genome sequences. Schematics of genes are shown according to the scale bar on top and colored according to their functional category based on the key. Multi-color shaded plot above each genome indicates the average NT similarity to the other genomes (range = 50–100%) as generated by Mauve

**Fig. 4:**
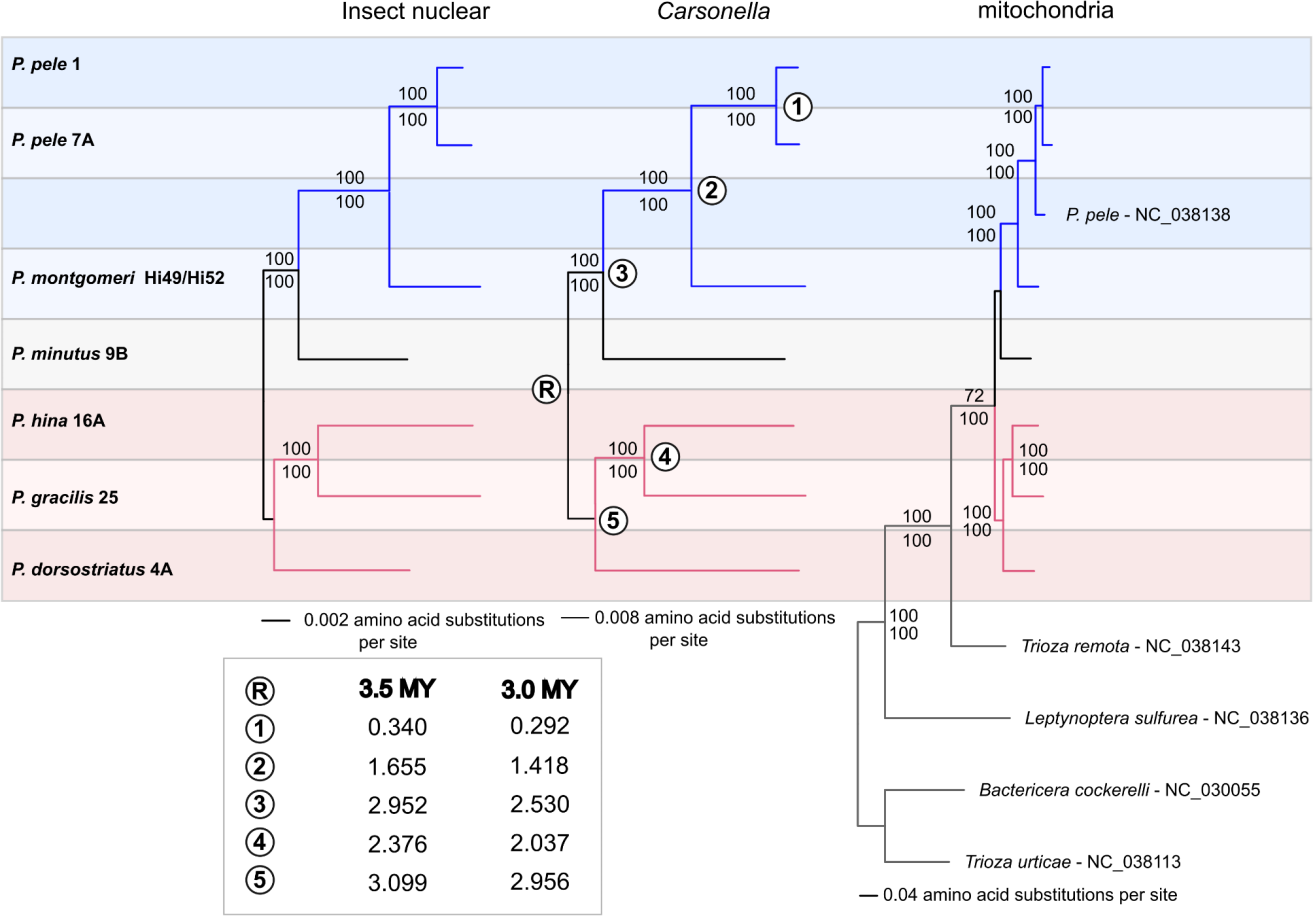
Core gene phylogenies of *Pariaconus* psyllids. Topological congruence detected among the best approximately-maximum-likelihood trees generated for the insect nuclear genes, *Carsonella* and entire mitogenome sequences. Branches are shaded by the major *Pariaconus* species groups *ohialoha* (blue), *minutus* (black), and *bicoloratus* (red) and sample datasets generated here are labeled to the left. An additional published *Pariaconus* mitogenome (Percy et al. 2018) and outgroup RefSeq mitogenomes from Triozidae psyllids (*n* = 4) are included in the mitogenome phylogeny. Adjacent to each node statistical support values ≥ 50 from FastTree are shown above and RAxML-ng bootstrap scores are shown below. Inset box shows estimated ages for nodes on the *Carsonella* phylogeny based on the split between the *bicoloratus* species group and the others occurring between 3 and 3.5MYA

Mitogenomes were assembled and annotated from six individual psyllid metagenomes and one transcriptome sample (Fig. 3). Overall, the mitochondrial genomes were highly similar as might be expected for mitochondria from members of the same genus. The genome assembly sizes ranged from 14,168 to 15,031 bp and the base composition averaged ~ 24.1 ± 0.6% G + C (± SD) (Supplemental Table 2). Like the previously sequenced *P. pele* mitochondrial reference genome in NCBI the seven new sequences encode the same 13 core proteins (ND2, COX1, COX2, ATP8, ATP6, COX3, ND3, ND5, ND4, ND4L, ND6, CYTB, ND1), two rRNAs and 22 tRNAs. This genetic organization is shared with other non-Hawaiian members of the Triozidae (e.g., *Bactericera cockerelli*) (Figs. 3 and 4) [27]. As expected, the control region upstream of the rRNAs proved the most difficult to assemble, due to the high A + T content and palindromic nature of this region and was not fully resolved in 5 of the 7 genomes. Regardless, the genomes are completely syntenic and there are no apparent chromosomal rearrangements or gene losses (Fig. 3). In addition, these genomes fall within the known diversity of the previously sampled marker gene data from *Pariaconus* species populations (Supplemental Fig. 1).

### Identification of Pariaconus nuclear core genes

The majority of the psyllid metagenome or transcriptome short read assemblies are derived from the insect’s nuclear genomic DNA or RNA transcripts respectively. Although the contig coverage is fairly high among the samples (e.g., read coverage average for single copy gene contigs = 489X ± 70 SD), the assemblies remain incomplete and fragmented. Without a completed *Pariaconus* genome available, we employed a conservative approach to detect *Pariaconus* single copy orthologs using BUSCO sequences characterized in the outgroup psyllid *B. cockerelli*. Ultimately, we identified 198 of 1,677 *B. cockerelli* single copy ortholog transcripts [24] present in all 7 *Pariaconus* metagenome or transcriptome assemblies (Supplemental Table 3). These genes represent a variety of core biological processes in Hemipterans including RNA metabolism (GO:0016070) and processing (GO:0006396), translation (GO:0006412), metabolism of aromatic (GO:0006725) and nitrogen (GO:0006807) compounds (Supplemental Table 3).

### Estimating the Pariaconus species tree

Despite distinct differences in their cell and tissue distributions within psyllids, *Carsonella* and mitochondrial genomes exhibit strict patterns of maternal (vertical) inheritance [38]. In consequence, whole genome phylogenetic reconstructions with the *Carsonella* and mitochondrial genomes conform to previously shown species relationships [8, 27] (Fig. 4). Furthermore, the psyllid host single copy core gene phylogeny also shares this tree topology (Fig. 4). Notably, there are now three complete mitochondrial genomes from *P. pele*, one is from the form *kohalensis* and two from form *pele.* The two from form *pele* are from genetically distinct groups that correspond to a clade of geographically isolated populations and a clade with a broad geographic distribution and mixing, otherwise the two clades are of similar age and genetic diversity [8] (Supplemental Fig. 1). Among the *P. pele* mitochondrial genomes the coding sequences have an average range of pairwise nucleotide similarity of 92–98.6%. Whereas, between *Pariaconus* species the mitochondrial coding sequences have an average range of pairwise nucleotide similarity of 75.5–87.6%.

We previously estimated the divergence dates for five of these psyllid species using the six available *Carsonella* genomes with information on their host distribution and the geological dates corresponding to the origins of the Hawaiian Islands [7, 8]. Using this same approach, we re-estimated the ages by including the *Carsonella-Pm* genome sequenced here (Fig. 4). The resulting date ranges are largely concordant with our previous estimates and suggest that *Carsonella-Pm* diverged from the lineage leading to *Carsonella-Pp* between 1.42 and 1.65 MYA. Together these date estimates allow us to examine the absolute evolutionary rates in *Pariaconus* symbiont genomes, as well as the insect host chromosome.

### Patterns of natural selection and mutation in Pariaconus and its vertically inherited intracellular genomes

Using estimated rates of synonymous and nonsynonymous substitutions, we observed overwhelming evidence of purifying selection acting on each of the genomes (dN/dS < 1). None of the insect, organelle, or symbiont genes (*n* = 359) examined have any overt evidence of positive selection acting on them (dN/dS > 1). Purifying selection appears strongest on the 13 mitochondrial genes (mean tree dN/dS 0.085 ± 0.011 SE), however we note that 11 of the 13 genes have evidence of saturated dS estimates (tree dS > 3). Regardless, each mitochondrial gene was evaluated for evidence of possible site-specific positive selection [33], however, none was detected. We found that the strength of purifying selection on the single copy core *Pariaconus* nuclear (mean tree dN/dS = 0.132 ± 0.008 SE, *n* = 195) and *Carsonella* (mean tree dN/dS 0.161 ± 0.010 SE, *n* = 151) genes were comparable. The additional symbionts detected in some *Pariaconus* species, *Makana* (*Morganella-like*) and *Malihini* (*Dickeya*-like) also experience genome-wide purifying selection (mean tree dN/dS 0.14 ± 0.005 SE and mean tree dN/dS 0.20 ± 0.004 respectively) [7]. A minority of genes in *Carsonella*,* Makana* and *Malihini* were previously identified to have signatures of site-specific positive selection [7], and only a single *Pariaconus* nuclear gene, the predicted mitochondrial small ribosomal subunit protein mRpL22 tested here exceeded the significance threshold for encoding potentially positive selected codon positions (2∆l > *χ*^*2*^1,5%). We note that ribosomal subunit proteins were among those detected previously in *Carsonella*,* Makana* and *Malihini* that also had potentially positive selected codon positions [7].

**Fig. 5:**
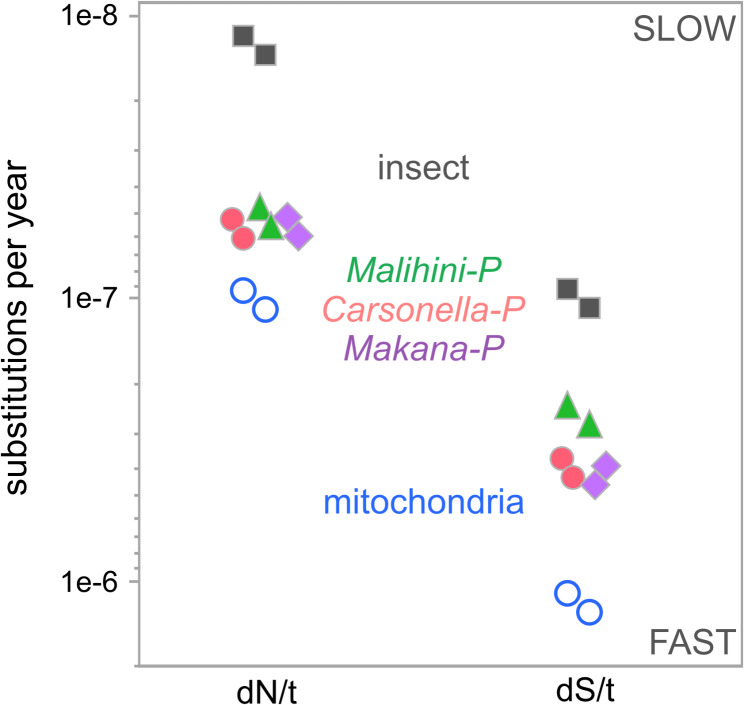
Evolutionary rates of *Pariaconus* psyllids and their associated genomes.Genome-wide estimates of time resolved rates of nonsynonymous (dN) and synonymous (dS) divergence in *Pariaconus* and its symbiont genomes. Two points are shown for the insect nuclear genes (black squares), *Carsonella* (red circles), *Makana* (purple diamonds), *Malihini* (green triangles), and the mitochondria (blue open circles) based on upper (3.5 MY) and lower (3 MY) bound time estimates

Although the mean estimated rates of dN/dS vary by less than 3-fold among the insect nuclear, mitochondrial and endosymbiont core gene sets, we find the underlying rates of normalized dN/t and dS/t can vary by as much as ~ 12-fold (Fig. 5). We find that the mere 13 protein coding sequences of the mitochondria have rates of dN/t and dS/t that are an average of 1.9X and 3.5X, respectively, greater than *Carsonella*,* Makana* and *Malihini*. Further, the mitochondria have average rates of dN/t and dS/t that are ~ 8.0X and 11.9X faster than the insect nuclear genes, respectively. These trends in *Pariaconus* and its intracellular genomes are consistent with what has been observed in other Hemipterans (e.g [6]).

### Rates of substitution in vertically inherited intracellular genomes

**Fig. 6:**
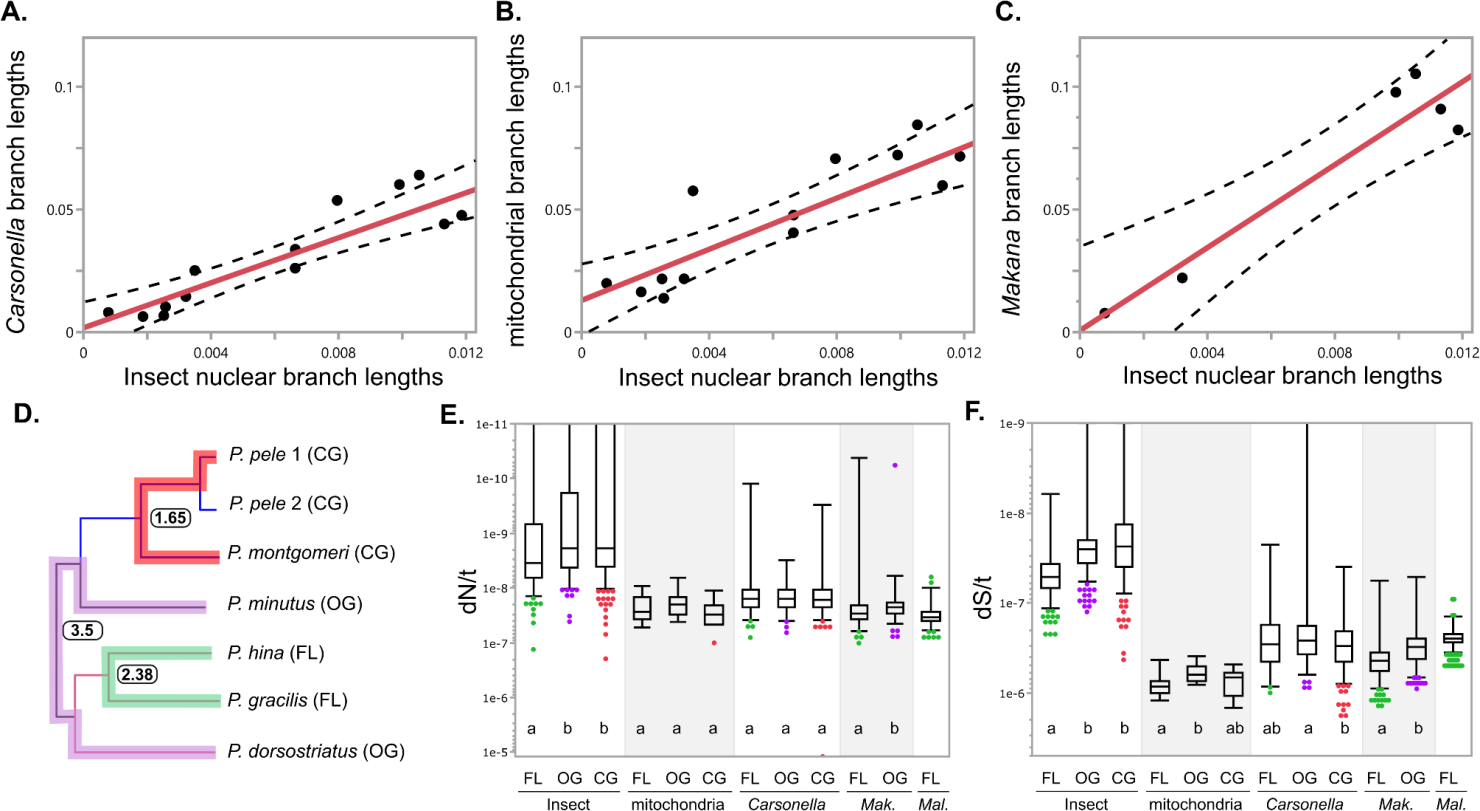
Lineage effects on evolutionary rates in *Pariaconus* psyllids. (**A-C**) Strong linear correlations detected among the branch lengths of amino acid based multi-gene phylogenies of the insect nuclear core genes, *Carsonella*, mitochondria, and *Makana* (Fig. 4, Pearson’s *R* ≥ 0.86 and *p* < 0.01). (**D-F**) Absolute rates of nonsynonymous and synonymous substitutions were compared among 3 pairs of genomes representing distinct galling patterns and number of intracellular symbionts. Species pairs are highlighted on the phylogeny and each with their maximal estimated age used to calculate dN/t and dS/t. Distributions of (**E**) dN/t and (**F**) dS/t are shown and letters indicate distinct statistical groupings based on nonparametric comparisons for each pair using the Wilcoxon Each Pair Method (*p* < 0.01)

In an initial assessment for correlated rates, we found significant correlations between the branch lengths of the whole proteome phylogenies for *Pariaconus*, *Carsonella*, the mitochondria and *Makana* (Fig. 6A-6). Each phylogeny was generated from a concatenated amino acid alignment of the single copy orthologs (Fig. 4). As observed above the topologies for *Pariaconus*, *Carsonella* and the mitochondria are identical (Fig. 4), and while *Makana* is not detected in all these psyllid species, it shares a co-phylogeny with the *bicoloratus* and *minutus* psyllids [7]. Despite some outlying branches, significant correlations among the shared branch lengths were detected when comparing *Pariaconus* with *Carsonella* (*R* = 0.907, F [1, 10] = 46.24, *p* < 0.0001), *Pariaconus* with the mitochondria (*R* = 0.864, F [1, 10] = 29.32, *p* = 0.0003), and *Pariaconus* with *Makana* (*R* = 0.951, F [1, 4] = 38.32, *p* = 0.0035). The third endosymbiont *Malihini* was not included in this analysis as it is only found in two of the seven *Pariaconus* samples we analyzed. However, these branch lengths are based on the estimated number of amino acid substitutions per site and do not address possible differences in underlying mutation rates (e.g., dS).

Therefore, we calculated pairwise estimates of dN and dS for *Pariaconus*, the mitochondria, *Carsonella*, and *Makana* from the three species pairs (free-living, 3 symbionts: *P. gracilis* vs. *P. hina*; open-gall, 2 symbionts: *P. minutus* vs. *P. dorsostriatus*; and closed-gall, 1 symbiont: *P. pele* vs. *P. montgomeri*) and normalized these values based on their divergence (e.g., dN/t, dS/t) (Fig. 6D). To be conservative, we used the predicted upper bound age estimates for the indicated nodes, assuming the earliest divergence of 3.5 MYA. Among the host nuclear genes, we detected significantly faster rates of dN/t and dS/t among the free-living psyllids (*P. gracilis* vs. *P. hina*) with three endosymbionts, *Carsonella*,* Makana*,* and Malihini*, on average ~ 1.6X, than open-gall psyllids (*P. minutus* vs. *P. dorsostriatus*) with two endosymbionts, *Carsonella* and *Makana*, and the closed-gall psyllids (*P. pele* vs. *P. montgomeri*) only with *Carsonella* (Fig. 6E & 6). This trend is partially explained by our observation that 7% of *P. minutus* vs. *P. dorsostriatus* and 30% of *P. pele* vs. *P. montgomeri* nuclear gene comparisons in fact have dN/t or dS/t measures of zero compared to only 3% of *P. gracilis* vs. *P. hina* genes. We note that this result is unlikely to be simply a result of sequencing/assembly error as the estimates of dN/dS are not significantly different among the host nuclear genes for all three pairs of species (Supplemental Fig. 2), but it does not rule out potential issues associated with allelic heterozygosity or the accuracy of our dating estimates. However, it can suggest that the *P. gracilis* vs. *P. hina* lineage is experiencing a slight but significant increase in underlying mutation rate and subsequent fixation of nonsynonymous substitutions.

Using these same dating estimates when comparing the mitochondrial and *Carsonella* genes we detected no significant differences in the estimates of dN/t between any of the pairs of genomes for any of the three psyllid species pairs (Fig. 6E). However, rates of dS/t exhibit some slight but significant variation with the species pairs ranked differently (Fig. 6F). For example, in open-gall psyllids (two symbionts) (*P. minutus* vs. *P. dorsostriatus*) mitochondrial genes have slightly slower average rates of dS/t (~ 1.4X) than the free-living psyllid species pair (two symbionts), and *Carsonella* genes have slightly slower average rates of dS/t (~ 1.3X) than the closed-gall psyllid species pair (one symbiont). It is worth noting that only 9–10 of the mitochondrial genes per species pair were compared as several dS estimates were saturated (dS > 3), whereas 123–150 non-saturated *Carsonella* genes were included in these analyses. Therefore, although mitochondrial and *Carsonella* genomes have notably faster mutation rates than the host nuclear genome (Fig. 5), we only detect subtle increases in dS/t for the free-living (*P. gracilis* vs. *P. hina*) species pair.

Further examination of the *Makana* genomes identified significant increases in dN/t (~ 1.2X) and dS/t (~ 1.4X) of the free-living species pair (two symbionts) (*P. gracilis* vs. *P. hina*) when compared to the *Makana* genomes from the open-gall species pair (one symbiont) (*P. minutus* vs. *P. dorsostriatus*) (Fig. 6E & 6). This again relied on the same dating estimates to calculate these rates. A systemic dating issue would be expected to affect all estimates uniformly. However, the lack of significant differences in the dN/t estimates between mitochondrial and *Carsonella* gene sets supports the possibility that the presence of three symbionts and/or the free-living habit of the *P. gracilis vs. P. hina* lineage may contribute to the elevated mutation rates and the subsequent fixation of nonsynonymous substitutions in these insects and their associated genomes.

Finally, despite the differences detected in dN/t we find strong, significant correlations in dN/t among gene pairs in all four data sets (Supplemental Fig. 3). The presence of correlated protein divergences suggests that despite potential variations in the global mutation rate or fixation of amino acid substitutions, the majority of the genes in these genomes continue to experience parallel selective constraints.

## Discussion

Island archipelagos provide unparalleled opportunities to study species evolution. Here we have interrogated the absolute rates of sequence change in the nuclear, mitochondrial, and co-evolved beneficial symbiont genomes of *Pariaconus* psyllids that have occurred during the last 3.5 MY as they radiated among the Hawaiian Islands. Contrary to previous reports of the decoupling of rates between mitochondria and symbiont genomes in some multi-symbiont insect-symbiont systems [5, 6], our findings reveal strong evidence of correlated substitution rates between these maternally transmitted genomes across the *Pariaconus* psyllid species tree (Fig. 6A-6), irrespective of insect lifestyle or the number of beneficial symbionts present. Further, we detected signatures of elevated (1.2–1.6X) rates of synonymous substitutions in all the genomes associated with the lineage of free-living psyllids *P. gracilis* and *P. hina*, which harbor the three co-evolved beneficial symbiont genomes *Carsonella*,* Makana*, and *Malihini* [7]. The elevated rate of synonymous substitutions, often used as a proxy for estimating mutation rates, appears to have also impacted the number of nonsynonymous substitutions in both the host’s nuclear genes and the symbiont *Makana* in these two psyllid species (Fig. 6E & 6). This suggests a potential for increased fixation of beneficial amino acid substitutions. Furthermore, validating previous studies [7, 8, 27] the phylogenetic reconstructions based on the newly assembled host nuclear genes, the *Carsonella-Pm* genome, and the reconstructed mitogenomes all converge on a single evolutionary history for this rapidly radiating group (Fig. 4).

### Genome-wide patterns of sequence divergence

Microbial transitions from free-living to host-associated, intracellular lifestyles have radical impacts on their patterns of genome evolution [39]. The loss of recombination and horizonal gene transfer, combined with intrinsic deletion biases, and repeated genetic bottlenecks generally leads to genome shrinkage, elevated rates of mutation and compositional biases [39, 40]. Moreover, the longer these processes go on, the more extreme and irreversible these changes are [41]. Thus, as expected the greatest inferred mutation rates (dS/t) were detected among the insect mitogenomes, which are organelles that are derived from intracellular symbionts that have evolved over the past 2.5 billion years. And despite the differences in their gene contents (e.g., DNA repair machinery), the *Pariaconus* symbionts *Carsonella*,* Makana*, and *Malihini* have very similar estimated mutation rates (Fig. 5) [7].

Here we provide strong evidence that mutation rates are correlated between the mitochondrial and symbiont genomes across the *Pariaconus* psyllid species in this recently diverged radiation, and that this correlation is independent of the number of symbiont partners or host lifestyles. In consequence, our findings for *Pariaconus* resemble those observed in mono-symbiont systems (such as *Blochmannia*-Carpenter Ants, *Blattabacterium*-Cockroaches, and *Buchnera*-Aphids) where mitochondrial and symbiont gene evolution rates have been shown to be correlated [5, 13, 14]. In contrast, widely divergent auchenorrhynchan multi-symbiont systems that have *Karelsulcia* and one or more partner symbionts, including the Hawaiian *Nesophrosyne* leafhoppers, exhibit a decoupling of rates [5, 6]. The authors suggest that this decoupling may arise because these symbionts have greater genetic autonomy from their hosts and mitochondria, particularly in terms of their population sizes, replication or repair rates [6]. For instance, in the *Nesophrosyne* leafhoppers that exhibit decoupled rates, its symbionts *Nasuia* and *Karelsulcia* are among the fastest and slowest evolving insect endosymbionts respectively [6]. Whereas, in *Pariaconus* all three symbiont groups have remarkably similar rates, despite different suites of replication and repair genes and predicted ages of their association with *Pariaconus* psyllids (Fig. 5) [7]. It still needs to be experimentally determined whether differences in symbiont population sizes, replication, or transmission contribute to the coupling or decoupling of these rates. 

Although the rates of symbiont and mitochondrial genomes in *Pariaconus* are correlated, we did find that the free-living psyllids with three symbionts have evidence of elevated mutation rates influencing their nuclear, mitochondrial, and symbiont genomes compared to the other psyllid groups examined. The underlying mechanisms contributing to this remain unclear. One key phenotypic difference between free-living and galling *Pariaconus* psyllids, which may be shaped by distinct mutation rates and/or selection pressures, is the greater within-group morphological divergence of immature stages in free-living species. In contrast, closed-gall species exhibit nearly identical immatures, while open-gall species show morphologically intermediate within-group divergence [8]. Potential selection pressures that could influence immature morphology differently and are related to the presence or absence of a galling habitat include microenvironmental stressors such as temperature, UV exposure, and moisture loss, as well as increased vulnerability to natural enemies, and the presence/absence of foraging behaviors to acquire sufficient nutritional resources [42, 43]. Further field studies that observe these latter mechanisms are needed to better understand why mutation rates may differ between free-living and galling *Pariaconus* psyllids.

### Errors in dating

Estimating divergence times is fraught with challenges [35]. However, when dating data are available, these estimates offer valuable insights into absolute rates of genomic change and have, for example, highlighted the remarkable rate at which some microbial endosymbionts can evolve [6]. Several factors can contribute to inaccuracies in estimated dates; however, in the results presented here we can rule out several factors from adversely influencing our findings. First, overt errors in the sequencing or assembly of the short reads are expected to introduce errors randomly within coding sequences. Given the relative frequency of nonsynonymous sites are twice as common as synonymous sites, sequencing or assembly errors should inflate dN estimates, resulting in dN/dS values closer to 1. However, we find that average dN/dS estimates are significantly less than 1 and that dN/dS distributions are not significantly different among the insect nuclear, *Carsonella* or mitochondrial genes for all three pairs of psyllid species. Note that sequence read coverage for these different genomes can vary by ~ 10 to 100-fold. This suggests that our transcriptome and metagenomes are not adversely impacted by sequencing or assembly errors. Second, we do not expect allelic heterozygosity to have a dramatic effect on our dating estimates either. While all the metagenomes were derived from single individuals, the transcriptome was from a pooled RNA sample. However, we see no patterns of bias associated with the *P. montgomeri* RNA transcriptome assembly. Finally, a systemic dating issue would be expected to affect all estimates in a uniform fashion. So, while we see elevated dS/t in all the associated genomes from the free-living pair of *Pariaconus* species we only see significant differences in the estimates of dN/t in the insect nuclear genes and the *Makana* endosymbiont.

### *Carsonella* of the *ohialoha* group

Sequenced *Carsonella* genomes are highly eroded compared to free-living bacteria, ranging in size from 148 to 176 kbp (NCBI 2024) with *Carsonella* of *Pariaconus* psyllids averaging around 152 kbp. Among *Pariaconus* psyllids of the *ohialoha* group, which contains the closed-gall psyllids, we only detect minimal changes in *Carsonella* gene content during the last 1.42–1.65 MY (Fig. 2). This resulted in the retention of the complete histidine biosynthesis, which is eroded or entirely absent from the *bicoloratus* group of *Pariaconus* psyllids, which contains both the open-gall and free-living psyllids. Overall, fewer changes in gene content are detected among the *ohialoha* (closed-gall only) and *minutus* group (open-gall only) *Carsonella* genomes than are detected among the *bicoloratus* group (free-living and open-gall) [7]. It is unclear what aspects of the insect diet, lifestyle (closed-gall, open-gall, free-living) or number of symbionts (1 vs. 2 vs. 3) may have contributed to these gene losses, particularly genes associated with the histidine pathway. However, we have previously speculated that an initial loss of genes involved in producing upstream intermediates for histidine biosynthesis (e.g., ribose-phosphate diphosphokinase (*prs*)) may have led to cascading *his* gene losses due to relaxed purifying selection [7]. Notably, this biosynthetic pathway is not supplemented or replaced in free-living *Pariaconus* psyllids by genes encoded in the other partner symbionts such as genes in the arginine and B-vitamin biosynthesis pathways that have been detected in *Makana* and *Malihini*, respectively [7]. Further, independent losses of histidine biosynthesis have been detected in *Carsonella* genomes from other psyllid families (Aphalaridae, Psyllidae) and some of these species make closed galls (e.g., *Pachypsylla spp.*) [44]. Therefore, lifestyle alone does not determine the loss of the histidine biosynthesis pathway; host plant and diet are also likely contributing factors. Regardless, with the absence of recombination or horizontal gene transfer, such gene losses in obligate intracellular endosymbionts are irrevocable and could constrain future evolutionary options for lineages that have lost these biosynthetic pathways.

### Mining metagenomes

Our insect metagenomic libraries were prepared from whole animal gDNA or RNA extractions therefore the symbiont derived nucleic acids generally only represents 3% or less of the total read data (Supplemental Table 1) [7]. Therefore, we analyzed these data for additional insights into the evolution of *Pariaconus*, successfully identifying mitogenomes for each sample, as well as a core set of insect nuclear genes. As we have noted, the basal divergence of our species trees occurred over 3 million years ago [8]. Yet, despite the rapid radiation and diversity of the *Pariaconus* species examined we observe complete synteny of their mitochondrial genomes. Within *P. pele*, one of the most densely sampled species, we observed an average mitochondrial gene divergence of ~ 6% compared to ~ 16% between *Pariaconus* species and ~ 31% to a non-Hawaiian Triozidae species. However, *Pariaconus* are still subject to significant purifying (negative) selection which is expected to maintain the functions of the mitochondrial encoded proteins. In addition, we were able to recover an entire mitochondrial genome from an RNAseq sample. As other researchers have noted that despite variance in read coverage, RNAseq data sets are a valuable resource for recovering novel mitochondrial genomes [45, 46].

While the set of insect nuclear genes we identified likely represents only a small fraction of the total *Pariaconus* proteome, these genes enabled us to corroborate the species relationships identified based on the *Carsonella* and mitogenome datasets. Furthermore, these nuclear genes suggest that changes in the estimated rates of substitution on the free-living *Pariaconus* lineage are impacting not only the symbiont genomes, but the nuclear genome as well.

## Conclusions

The Hawaiian *Pariaconus* psyllid radiation is an ideal model to examine the intersection of recent species radiations with the evolving roles of intracellular endosymbiont partners. Our metagenomic analyses have produced a wide array of genomic markers for this taxon, its mitochondria and its bevy of intracellular symbionts that provide a better understanding of how these insects are being shaped by mutation, drift and natural selection. These data suggest that in *Pariaconus*, the presence of multiple endosymbiont partners does not lead to decoupled rates of sequence evolution. However, among psyllids that are free-living and have all three endosymbionts we did detect overall increases in the rates of substitution in the host and one of the endosymbiotic partners. Further metagenomic sequencing of additional species will be important for assessing the robustness of observed patterns in symbiont function and evolution within this clade. In particular, analyzing the basal *kamua* species group will be crucial for gaining a more comprehensive understanding of this psyllid radiation (Supplemental Fig. 1).

## Electronic supplementary material

Below is the link to the electronic supplementary material.

Supplementary Material 1

Supplementary Material 2

## Data Availability

The assembled mitochondrial genomes are available in the GenBank database of NCBI (https://www.ncbi.nlm.nih.gov/) under accession numbers PQ124090– PQ124096. DNAseq-based assemblies correspond to BioProject PRJNA1145550 and PRJNA1125431, BioSample IDs SAMN42896904– SAMN42896909 and SAMN47004634, and SRA accessions SRR30166534– SRR30166539. The RNAseq-based assembly was generated from BioSample ID SAMN04101425 and transcriptome data SRA accession SRR2496664. A more detailed description of the bioinformatic applications and commands used in these analyses, several custom scripts, and alignments of the insect core genes are available at: https://github.com/phdegnan/Evolution-in-Pariaconus.
