# Supplementary material for: Coupled evolutionary rates shape a Hawaiian insect-symbiont system
Source: BMC Genomics. 2025 Apr 3;26:336. doi: 10.1186/s12864-025-11514-z (PMC11970012; doi:10.1186/s12864-025-11514-z)
Supplement: Supplementary file 2 — Supplementary Material 2 [file 12864_2025_11514_MOESM2_ESM.pdf]

Title: Coupled evolutionary rates shape a Hawaiian insect-symbiont system

Patrick H. Degnan[1*], Diana M. Percy[2], and Allison K. Hansen[3*]

[1]Department of Microbiology and Plant Pathology, University of California, Riverside, California, USA.

[2]Department of Botany, University of British Columbia, Vancouver, BC, Canada

[3]Department of Entomology, University of California, Riverside, California, USA.

*Corresponding authors

Patrick H. Degnan[1] and Allison K. Hansen[3]

Email[1]: patrickd@ucr.edu

Email[2]: allison.hansen@ucr.edu

PHD ORCID: 0009-0007-0689-071X
DMP ORCID: 0000-0002-0468-2892
AKH ORCID: 0000-0002-3066-5527

Supplemental Figures

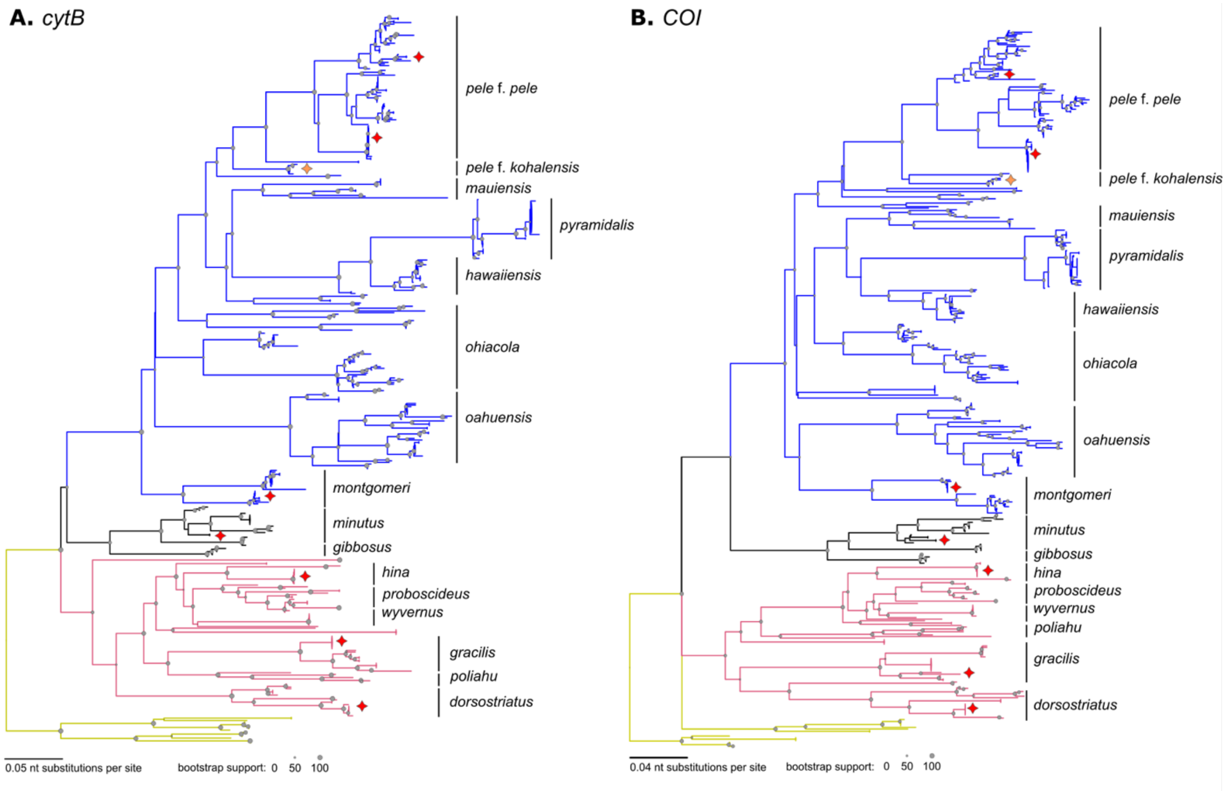

Supplemental Fig. 1 CYTB and COI gene phylogenies among described *Pariaconus* species. Best approximately-maximum-likelihood tree generated for (**A**) *cytB* and (**B**) *COI* fragments of GenBank deposited *Pariaconus* PCR amplified fragments (*n*=449) and an existing *P. pele* mitogenome (orange star), with the addition of new data from mitochondrial genome sequences (red stars). Branches are colored by the major species groups *ohialoha* (blue), *minutus* (black), *bicoloratus* (red) and *kamua* (yellow). Note, only densely sampled species are labeled, 12 other species are included but not labeled here due to space constraints. Bootstrap support values for nodes are shown by grey circles that are sized according to the scale.

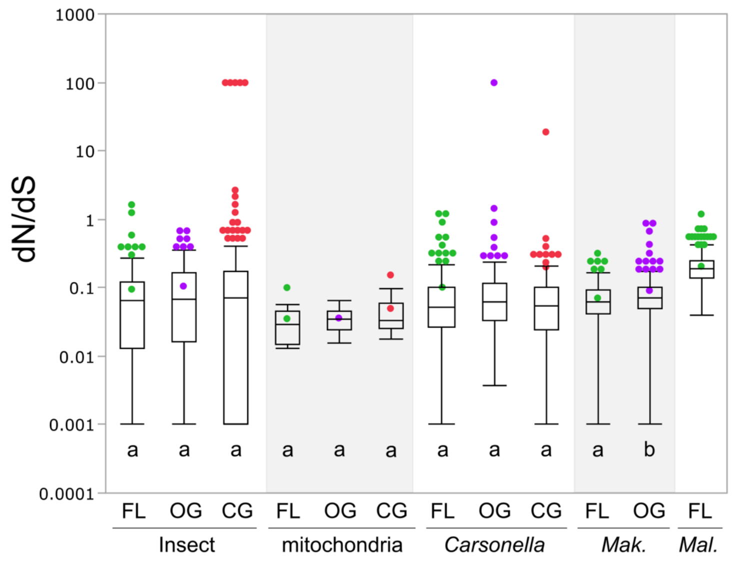

Supplemental Fig. 2. Purifying selection acts on *Pariaconus* psyllids and their associated genomes. Pairwise estimates of natural selection acting on each gene were calculated in PAML (number of nonsynonymous substitutions per nonsynonymous sites divided by the number of synonymous substitutions per synonymous sites) among 3 pairs of genomes representing distinct galling patterns and number of intracellular symbionts (see **Figure 6**). Letters indicate distinct statistical groupings based on nonparametric comparisons for each pair using the Wilcoxon Method ($p < 0.01$).

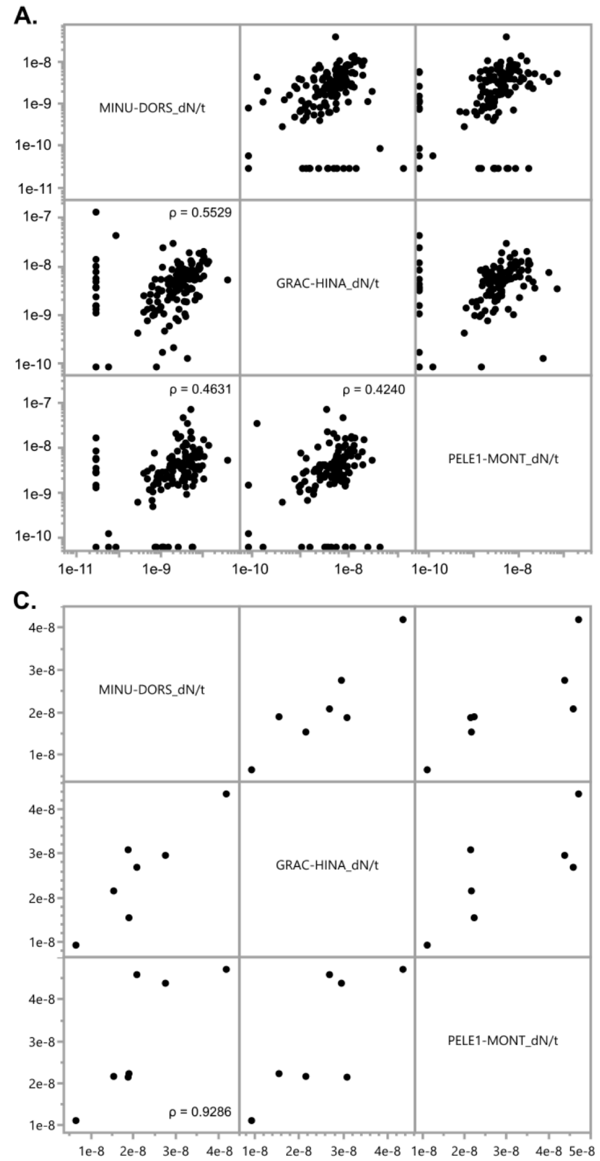

Supplemental Fig. 3. Correlated rates of nonsynonymous substitutions in *Pariaconus* psyllids and their associated genomes. Nonparametric correlations (Spearman's rho, ρ) were calculated for pairwise estimates of nonsynonymous substitutions per nonsynonymous sites for each gene in the (**A**) insect nuclear (**B**) *Carsonella*, (**C**) mitochondrial and (**D**) *Makana* datasets. All significant correlations (p < 0.01) are displayed in the appropriate quadrant.
